# Comparison of outcome between nonoperative and operative treatment of medial epicondyle fractures

**DOI:** 10.1080/17453674.2020.1832312

**Published:** 2020-10-19

**Authors:** Petra Grahn, Tero Hämäläinen, Yrjänä Nietosvaara, Matti Ahonen

**Affiliations:** a Department of Pediatric Orthopedics and Traumatology, New Children’s Hospital, HUS Helsinki University Hospital, Helsinki;; b Department of Orthopedics and Traumatology, HUS Helsinki University Hospital, Helsinki;; c Department of Pediatric surgery, Kuopio University Hospital, Kuopio, Finland

## Abstract

Background and purpose — Controversy exists regarding the optimal treatment for displaced medial epicondyle fractures. We compared the results of nonoperative and operative treatment and calculated the incidence of medial epicondyle fractures in the pediatric census population.

Patients and methods — 112 children under 16 years old who sustained > 2 mm displaced fracture of the medial epicondyle were treated in our institution between 2014 and 2019. 80/83 patients with 81 non-incarcerated fractures were available for minimum 1-year follow-up. 41 fractures were treated with immobilization only, 40 by open reduction and internal fixation, according to the preference of the attending surgeon. Outcome was assessed at mean 2.6 years (1–6) from injury with different patient-reported outcome measures. Elbow stability, range of motion, grip strength, and distal sensation were registered in 74/80 patients. Incidence was calculated for 7- to 15-year-olds.

Results — Nonoperatively treated children had less pain according to the PedsQL Pediatric Pain Questionnaire (3 vs. 15, p = 0.01) with better cosmetic outcome (VAS 95 vs. 87, p = 0.007). There was no statistically significant difference between the groups in respect of QuickDASH, PedsQL generic core scale, Mayo Elbow Performance Score, grip strength, carrying angle, elbow stability, or range of motion (p > 0.05). All 41 nonoperatively treated children returned to pre-injury sports; of the surgically treated 6/40 had to down-scale their sporting activities. The incidence of displaced (> 2 mm) fractures of the medial epicondyle in children aged 7–15 years was ≥ 3:100,000.

Interpretation — Displaced fractures of the medial humeral epicondyle in children heal well with 3–4 weeks’ immobilization. Open reduction and screw fixation does not improve outcome.

Fractures of the medial humeral epicondyle have been reported to account for 12–20% of all pediatric elbow fractures, but the incidence is not known. Elbow dislocation is associated with 30–50% of these fractures (Gottschalk et al. 2012), with an incarceration rate of the fracture fragment into the elbow joint of 5–18%. Ulnar nerve lesions are registered in 10–16% of cases (Louhaem et al. 2010).

Nonoperative treatment is advised in minimally displaced (< 2 mm) fractures of the medial humeral epicondyle, whereas surgery is recommended for fractures incarcerated in the elbow joint as well as for fractures that are either grossly unstable or where the ulnar nerve is entrapped (Smith 1950, Blount 1955, Maylahn and Fahey 1958, Bede et al. 1975, Gottschalk et al. 2012, Tarollo et al. 2015). Significant controversy concerning the treatment of displaced (3–15 mm) fractures exists, with some surgeons advocating early mobilization, some immobilization, and some internal fixation (Lee et al. 2005, Hughes et al. 2019, Pezzutti et al. 2020). It has also been suggested that competitive athletes or fractures occurring in combination with elbow dislocation should be treated surgically with a lower threshold than in children without sporting activities (Baety and Kasser 2014).

The reported outcome of nonoperative and operative treatment in displaced fractures of the medial humeral epicondyle in terms of elbow function and complications has been similar (Farsetti et al. 2001, Biggers et al. 2015, Axibal et al. 2019).

We compared subjective and objective outcomes and calculated the incidence of medial humeral epicondyle fractures in children treated either with immobilization or with open reduction and internal fixation (ORIF).

## Patients and methods

We conducted a controlled treatment trial based on prospectively collected data from consecutive patients identified from our institutional fracture registry. 112 (62 female) less than 16-year-old children who had sustained a more than 2 mm displaced fracture of the medial humeral epicondyle (modified ICD-10 code: S42.45) were treated in our tertiary level teaching hospital during a 6-year-long study period between January 2014 and December 2019. The incidence of displaced fractures of the medial humeral epicondyle was calculated in 7- to 15-year-olds in the catchment area, as nearly all children (109/112) who had sustained a fracture of the medial epicondyle were older than 6 years.

Mean age of patients was 12 years (4–16). 34/112 (30%) patients had a trampoline injury. Patients with the medial epicondyle incarcerated in the elbow joint (n = 9) and patients with less than 1-year follow-up (n = 20) were excluded from the outcome study. All patients with partial avulsions (n = 4) were excluded from both studies. 81/83 remaining children with 82 fractures could be contacted and were available for follow-up (FU).

**Figure 1.**
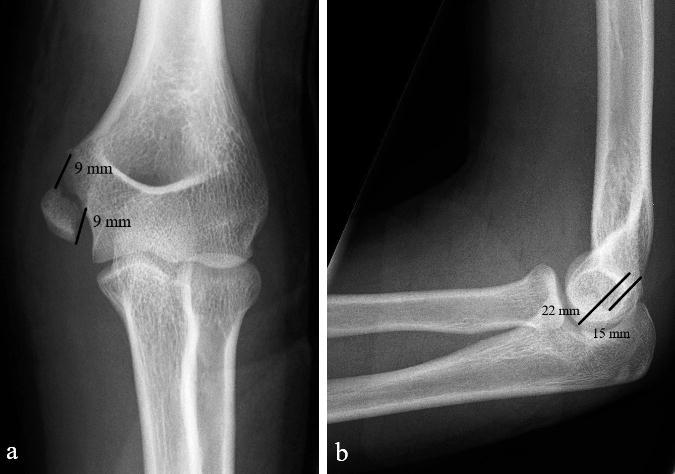
Medial epicondyle avulsion with displacement measures as described by Edmonds et al. (2010).

Treatment method was chosen by the attending surgeon’s preference and cases were not uniformly presented in a consensus conference. 41 of the patients available for FU had been treated primarily nonoperatively and 40 by ORIF. 1 primarily nonoperatively treated patient underwent reduction and screw fixation of a malunited fracture due to pain under load 5 months from injury; this outcome data is not included in the group analysis. Patients medial elbow pain continued why the fixation screw together with a hypertrophic scar were removed at 9 months postoperative. At the last FU 1 year from surgery she still had pain under load. There was little difference in sex distribution (24/41 vs. 20/40 male patients) and mechanism of injury between the nonoperatively and surgically treated children (Table 1, see Supplementary data). Fracture displacement was calculated by the method described by Edmonds (2010) in 50 cases from AP radiographs only and in the remaining 31 radiographs from both AP and lateral view (Figure 1). The mean fracture displacement in nonoperatively treated patients measured from AP radiographs was 8 mm (3–12) vs. 7 mm (4–13) in the surgically treated children and from the lateral view respectively (n = 17 and 16) 9 mm (6–22) vs. 9 mm (6–16). 2 of the 3 patients with less than 5 mm of fracture displacement were treated nonoperatively. Nonoperatively treated patients were somewhat younger with a mean age of 11 years (4–16) vs. 12 (7–16) for the surgically treated, and their fractures were more often associated with clinically or radiologically documented elbow dislocation (19/41 vs. 10/40).

**Table 2.** Results of patient-reported outcome measures at last follow-up. Values are mean (range), SD, and [95% confidence interval]

	Non-operative	ORIF
Score	n = 41 **^a^**	n = 40
Quick Dash	1.8 (0–13.6) SD 4 [0.1–2.9]	4.2 (0–23) SD 6 [2.5–5.9]
Quick Dash hobby module PedsQL	0.5 (0–6.3) SD 2 [0.0–1.1]	6.7 (0–100) SD 21 [0.3–13]
total score	89 (74–100) SD 9 [86–92]	90 (74–100) SD 7 [88–92]
physical functioning		
health summary score	93 (75–100) SD 7 [90–95]	91 (75–100) SD 7 [89–93]
pain module, VAS (0–100) **^b^**	3.3 (0–44) SD 8 [0.9–5.7]	15 (0–85) SD 22 [8–22]
Mayo Elbow Performance score	99 (85–100) SD 3 [98–100]	95 (80–100) SD 8 [93–97]
Cosmetic score, VAS (0–100)	95 (64–100) SD 9 [92–98]	86 (35–100) SD 17 [81–92]

**^a^** 1 bilateral

**^b^** PedsQL Pain module score represents the worst pain the patient has experienced in the injured elbow during the last 7 days.

Nonoperative treatment was carried out by immobilizing the injured upper extremity (1 bilateral injury) either with an above-elbow cast (n = 38) or a collar-and-cuff sling (n = 3) for a mean 24 days (19–34). Closed reduction was not attempted in the nonoperative group. Internal fixation was performed at mean 5 days (0–19) from the injury by a cannulated screw in 33, smooth pins in 6, or with a bone anchor in 1 patient. Bone anchors were additionally used in 2 instances, once in combination with a screw and once with pins. Half of the operations were done by an attending pediatric orthopedic surgeon or a pediatric surgeon, half by registrars. Mean length of postoperative immobilization was 30 days (21–44) either in an above-elbow splint (n = 36) or with a collar-and-cuff sling (n = 5). All wounds healed uneventfully without recorded infections. The rate and timing of hardware removal was registered.

39 of the nonoperatively treated children were examined in our outpatient clinic and 2 interviewed by phone at mean 2.8 years (1–5) from the injury against 36 of the operated children, with 4 interviewed by phone at 2.4 years (1–6). Subjective outcome was assessed in 80 patients with QuickDASH (Beaton et al. 2005), Pediatric Quality of Life InventoryTM (PedsQL) Generic Core Scale, PedsQL Pediatric Pain Questionnaire (Varni et al. 1999), Mayo Elbow Performance Score (Morrey 1993), as well as a cosmetic visual analogue scale (VAS 0–100). Patients interviewed by phone were asked to answer the PedsQL Pain Questionnaire, Cosmetic VAS, and QuickDASH main and hobby module. Patients’ pre- and post-injury participation in non-organized and organized sports was registered. In the outpatient clinic carrying angle, and active and passive range of motion (ROM) of both elbows was measured using a goniometer. Stability of the elbow was assessed by the moving valgus test (O’Driscoll et al. 2005) and the valgus stress test (Flynn et al. 2008). Grip strength of both hands was recorded as the mean of 3 efforts with a hydraulic hand-held dynamometer. Distal sensation was examined by Semmes–Weinstein monofilaments (Bell-Krotoski 1990). Prevalence of cold intolerance was assessed. 

### ^Statistics^

Data was analyzed using the Wilcoxon rank-sum test in Python 3.8 (Python Software Foundation, Wilmington, DE, USA). Our null hypothesis was that there is no difference in outcome between nonoperatively and operatively treated patients. Level of significance was set at p < 0.05. 

### ^Ethics, funding, and potential conflict of interest:^

Hospital ethical board approval was received in 1999. Extension permission for the study was approved on December 17, 2015 (approval number HUS 621/1999). None of the authors received any funding for the study and none of the authors report any conflict of interest.  

## Results

All patients completed the requested follow-up forms. There was no statistically significant difference in the QuickDASH, QuickDASH hobby module, PedsQL Physio Social Health Summary Score, PedsQL Physical Functioning Health Summary Score, PedsQL Total Score, or Mayo Elbow Performance Score. However, nonoperatively treated children had less pain according to the PedsQL Pediatric Pain Questionnaire (3 vs. 15, p = 0.01) with better cosmetic outcome (VAS 95 vs. 87, p = 0.007). For carrying angle, elbow stability, extension deficiency, flexion deficiency, active or passive ROM and grip strength, we discovered no statistically significant differences between uninjured and injured side within the group or between the groups (Table 2). A separate analysis excluding the 3 patients with < 5 mm displacement yielded the same results.

All examined patients had normal sensation in the pulp of their fingers as measured by Semmes–Weinstein monofilament test, but 1 operatively treated child interviewed by phone reported diminished sensation in the ulnar fingers. Another surgically treated patient had decreased sensation in a 5 x 6 cm area distal to the scar. Cold intolerance was reported by 1 nonoperatively and 2 surgically treated patients. Pain at the medial humeral epicondyle either with direct contact or under load was reported by 4 nonoperatively and by 6 operatively treated children with otherwise normal sensation and elbow stability (Table 3). All non-operatively treated patients had returned to the same or higher level of sport as pre-injury, whereas 6 surgically treated patients had downgraded their sporting activities (Table 1, see Supplementary data).

**Table 3.** Results of clinical examination at last follow-up. Values are mean (range), SD, and [95% confidence interval]. Patients interviewed by phone excluded (n = 6).

	Non-operative	ORIF
Outcome	n = 39 **^a^**	n = 36
Carrying angle difference (°)	0.9 (0–8) SD 3 [0.3–1.8]	1.1 (0–5) SD 2 [0.4–1.9]
Extension deficiency (°)	1.0 (0–15) SD 4 [–0.3 to 2.3]	3.0 (0–20) SD 6 [1.0–5.0]
Flexion deficiency (°)	1.6 (0–10) SD 5 [0.1–3.1]	2.1 (0–15) SD 4 [0.9–3.3]
Valgus stress test	3 unstable without pain	1 unstable without pain
	2 stable with pain	4 stable with pain
	1 unstable with pain	
Moving valgus test	7 pain	9 pain

**^a^** 1 bilateral

**Figure 2.**
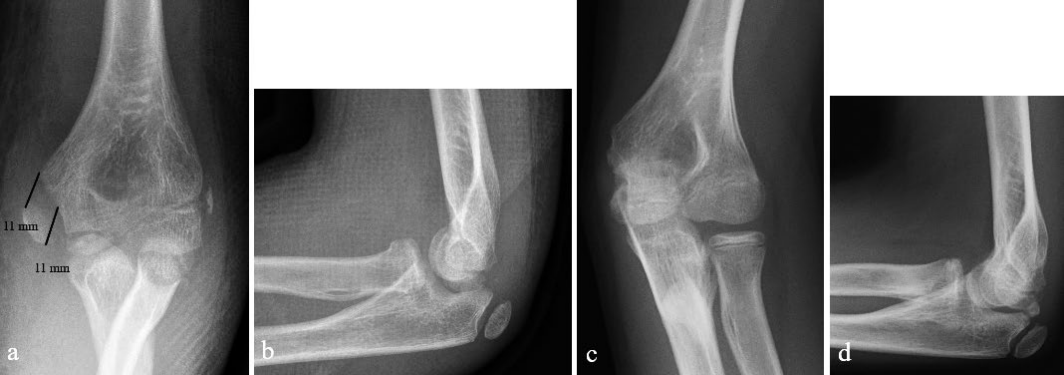
a, b. 11.8-year-old boy with 11 mm displaced fracture of the medial humeral epicondyle, which was treated with an above-elbow splint for 3 weeks. c, d. He had returned to climbing without pain and his elbow was stable with a full range of motion at 4 years from injury.

**Figure 3.**
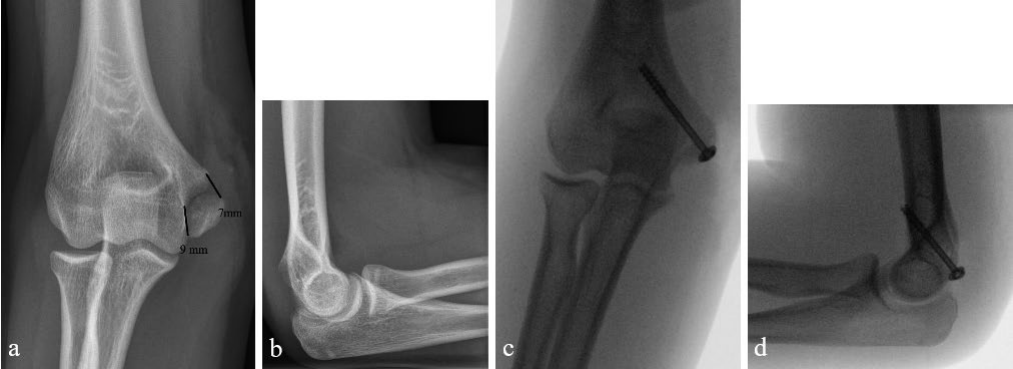
a, b. 12.6-year-old gymnast's 8 mm displaced fracture of the medial humeral epicondyle, which was anatomically reduced and fixed with a well-positioned 4 × 45 mm cannulated screw. c, d. At follow-up 3 years from injury, she had returned to competitive gymnastics. She reported no pain and she had no functional problems, although her valgus stress test was positive (unstable without pain).

Pins were removed in the outpatient clinic from 5/6 children who had had their fractures pin fixed. 10 of the 33 children who had their fractures fixed with a cannulated screw had had their screws removed due to local pain at mean 16 months (7–29) from the injury. 6 children’s fractures were fixed in malposition (4 with screws, 2 with pins), but 5 of these 6 children were pain free with normal elbow stability and function. The training level of the operating surgeon did not affect the outcome assessed by the different PROMs used (p > 0.05, Figures 2–3).

Between 2014 and 2019, 525,966 children between the ages of 7–15 years lived in the catchment area (national registry). During the same period 76 children from the same area in the same age range had a displaced (> 2mm) fracture of the medial humeral epicondyle, giving a mean annual incidence of 3:100,000. The peak incidence occurred at 11 years of age. The real incidence may be slightly higher, as 10 additional children’s medial epicondyle fractures (city of residence unknown) had been treated in private clinics according to a survey conducted among our region’s pediatric orthopedic surgeons. 

## Discussion

Operative treatment of pediatric medial epicondyle fracture has gained popularity, although there is little evidence in support of surgical treatment over nonoperative. Decision to operate is often based on the degree of displacement and mechanism of injury. Surgery is often recommended in displaced fractures and in fractures sustained in association with elbow dislocation (Blount 1955, Maylahn and Fahey 1958, Vecsei et al. 1975, Josefsson and Danielsson 1986, Farsetti et al. 2001, Lee et al. 2005, Lawrence et al. 2013, Pezzutti et al. 2020).

Currently there is no consensus on how fracture displacement should be measured or how to define a clinically significant fracture displacement. Edmonds et al. (2010) have shown that measurements from plain radiographs are unreliable, and argued that computer tomography (CT) should be used. Some authors advocate surgery in fractures with as little as 3 mm displacement (Vescei et al. 1975, Baety and Kasser 2014, Hughes et al. 2019), while most surgeons would consider operative treatment if it exceeds 5 mm (Smith 1950, Pezzutti et al. 2020). In most of our patients’ plain radiographs fracture displacement could be measured only from the AP view and the actual displacement could thus be bigger. More reliable measurements could have been made from CT scans, which were not routinely taken. Our results suggest, however, that CT is unnecessary as the degree of fracture displacement does not seem to affect the outcome.

Louahem et al. (2010) argued that damage to the main medial stabilizer of the elbow, the medial collateral ligament, has far greater influence on elbow joint stability and outcome than actual fracture displacement and recommended surgery in patients with a positive valgus stress test, regardless of amount of fracture displacement. We did not routinely examine elbow stability in our patients at time of injury, an examination that often requires sedation. At follow-up there was no difference between the 2 treatment groups regarding stability of the elbow under valgus load. Nearly half of the nonoperatively treated children had an elbow dislocation, which was a clearly higher rate than one-fourth in the surgically treated children, thus one could argue that good results in the nonoperatively treated group could in part be due to an intact ulnar collateral ligament as suggested by Gottschalk et al. (2012).

9 of the 42 children with a medial epicondyle fracture who were treated nonoperatively in the series of Smith et al. (2010), developed a symptomatic nonunion in a 1-year follow-up. Contributing factors to the cause of the pain were not found. On the other hand, none of 139 surgically treated patients reported pain at mean 3.9 years follow-up in the series of Louhaem et al. (2009). However, Axibal et al. (2018) found no difference in elbow pain according to a phone survey at minimum 1.5 years after a medial epicondyle fracture between 28 patients treated with cast immobilization and 14 operatively treated children. Our findings contradict these previous reports because our nonoperatively treated patients had less pain than the operatively treated children. We also found that nonoperatively treated children were more pleased with the appearance of their injured elbow than children who had undergone surgery.

There is little information concerning return to sport in children who have sustained a fracture of the medial humeral epicondyle. According to the study by Lawrence et al. (2013) there was no difference in outcome assessed by QuickDASH and elbow range of motion at 2 years from injury in 6 nonoperatively and 14 operatively treated athletes. Axibal et al. (2018) showed similar results with no difference in the objective outcome in less than 1-year follow-up between 22 operated patients matched with 22 nonoperated patients. All nonoperatively treated children returned to their previous sports, whereas 6 of the operatively treated patients could not continue their sports at all or returned to a lower level. It thus appears that the rate of return to sports cannot be improved by open reduction and pin or screw fixation of the fractured medial humeral epicondyle. This finding should be interpreted with caution, since patients were younger in the nonoperative group than in the operatively treated group.

Medial humeral epicondyle fractures are reported to represented 1/5 of elbow fractures in children (Gottschalk et al. 2012, Baety et al. 2010). To our knowledge, the incidence of humeral medial epicondyle fractures in the pediatric census population has not been reported. We chose to calculate and report the incidence for 7- to 15-year-olds (≥ 3:100,000 in the catchment area) rather than for the entire pediatric population as 99% of patients with medial epicondyle fracture in the study population were 7 years or older. The most common injury mechanism was trampoline, followed by falls from height often associated with cheerleading or different types of gymnastics (Table 1, see Supplementary data).

There are few prospective and no randomized controlled treatment trials for fractures of the medial humeral epicondyle in children. Most published studies represent small retrospective hospital-based patient series. The degree of fracture displacement or the stability of the elbow before commencing treatment is seldom registered.

### ^Strengths and limitations^

This is a comparative study of 81 consecutive children prospectively collected who had sustained a > 2 mm displaced medial epicondyle fracture treated by surgeon’s preference either by immobilization or by ORIF with a high follow-up rate, 81/83. Treatment was not randomized, which may cause a bias. Mean age of patients in the nonoperative group was lower than in the ORIF group. We do not have an obvious explanation for this discrepancy, but in general younger children less often require operative treatment in pediatric orthopedic trauma, which may have had an effect on selecting treatment modality. CTs had not been taken routinely and the exact fracture displacement could not therefore be measured. Regardless of treatment some patients remain symptomatic under valgus stress. This raises the question as to whether our treatment decisions are based on the right parameters, e.g., displacement of the fracture fragment vs. medial collateral ligament injury. In light of the shortcomings of this study we have been granted ethical review board permission to start a randomized control trial conducted as a non-inferiority trial.

In summary, non-incarcerated fractures of the medial humeral epicondyle in children and adolescents can be safely and reliably treated nonoperatively with 3–4 weeks’ cast immobilization. The degree of primary fracture displacement or elbow dislocation does not seem to affect the outcome, since normal elbow functions are restored with few exceptions.  

## Supplementary Material

Supplemental Material

## Supplementary data

Table 1 is available as supplementary data in the online version of this article, http://dx.doi.org/10.1080/17453674.2020.1832312
