# Supplementary material for: Comparison of outcome between nonoperative and operative treatment of medial epicondyle fractures
Source: Acta Orthop. 2020 Oct 19;92(1):114–9. doi: 10.1080/17453674.2020.1832312 (PMC7919892; doi:10.1080/17453674.2020.1832312)
Supplement: Supplemental Material [file IORT_A_1832312_SM6755.pdf]

Supplementary data

Table 1. Mechanism of injury and participation in organized sport before and after injury

Non-operatively treated						Open reduction internal fixation					
Age at injury	Dominant side injury	Elbow dislocation	Mechanism of injury	Follow-up (years)	Organized sports	Age at injury	Dominant side injury	Elbow dislocation	Mechanism of injury	Follow-up (years)	Organized sports
4.3			Fall from height	1.2	No	7.5	Yes		Fall from height	2.6	No
5.0	Yes	Yes	Fall at same level	2.5	Artistic gymnastics	8.7 [a]	Yes		Fall from height	1.0	Floorball
6.6	Yes		Trampoline	1.2	Icehockey	9.4	Yes		Alpine skiing	2.1	Alpine skiing
6.7	Yes		Fall from height	5.0	Football	10.1	Yes		Trampoline	3.9	No
7.2			Fall from height	2.6	Riding	10.3 [a]	Yes		Fall from same level	1.1	Ballet
7.7			Fall at same level	3.6	Floorball	10.5 [a, d]	Yes	Yes	Trampoline	3.5	Basketball
8.0	Yes		Fall from height	3.9	No	10.6	Yes		Gymnastics	1.2	Artistic gymnastics
8.4	Yes	Yes	Trampoline	4.7	Trampoline gymnastics	11.0	Yes		Fall at same level	1.6	No
8.6			Hit by car (<20km/h)	5.2	Basketball	11.0 [a]			Cheerleading	1.9	Cheerleading
8.7	Yes		Fall from height	2.7	Volleyball	11.1	Yes	Yes	Gymnastics	2.3	Football
8.8			Trampoline	2.6	Football	11.1 [a]	Yes		Trampoline	1.0	Artistic gymnastics
8.9		Yes	Fall from height	3.5	Cheerleading	11.2 [d]	Yes		Football	1.4	Football (goalkeeper)
9.4	Yes	Yes	Football	3.6	Football	11.3			Fall from height	1.9	Tennis
9.7	Yes	Yes	Riding	2.9	Riding	11.4	Yes		Artistic gymnastics	1.1	Artistic gymnastics
10.0			Fall at same level	1.5	Dance	11.7	Yes	Yes	Trampoline	2.1	No
10.1		Yes	Trampoline	3.5	Trampoline gymnastics	11.7			Bike	1.1	No
10.2			Cheerleading	1.3	Cheerleading	11.8	Yes		Alpine skiing	3.2	Handball
10.6		Yes	Trampoline	1.8	Cheerleading	11.9	Yes		Gymnastics	1.7	Cheerleading
10.6	bilateral	Yes	Trampoline	1.7	Karate	12.1	Yes		Fall at same level	1.1	No
10.7	Yes		Trick biking	2.8	Football	12.3	Yes		Judo	2.2	Judo
10.8	Yes		Judo	3.1	Judo	12.4			Gymnastics	1.0	Artistic gymnastics
10.8	Yes	Yes	Trampoline	1.3	Football	12.4	Yes	Yes	Trampoline	2.7	Taekwando
11.3			Trampoline	3.7	Football	12.6	Yes		Trampoline	2.9	Piano
11.8		Yes	Fall at same level	3.7	Climbing	12.6	Yes		Fall from height	1.5	Icehockey
11.9	Yes	Yes	Circus acrobatics	4.4	Cirkus acrobatics	12.7			Trampoline	1.0	Cheerleading
11.9	Yes		Arm wrestling	3.6	Athletics	12.7	Yes		Bike	2.9	Swimming
12.0			Cheerleading	4.2	Cheerleading	12.9 [a]			Trampoline	6.3	Football
12.0			Figure skating	1.0	Figure skating	13.0 [b]			Trampoline	1.2	Cheerleading
12.0			Alpine Skiing	1.1	Football (goalkeeper)	13.3			Bike	2.6	Football
12.2	Yes		Artistic gymnastics	3.9	Artistic gymnastics	13.5 [c]	Yes		Judo	1.0	Judo
12.4	Yes	Yes	Trampoline	2.4	Artistic gymnastics	13.5	Yes		Ringette	2.5	Ringette
12.4	Yes	Yes	Bouldering	1.2	Football	14.1 [d]	Yes		Fall from height	1.7	Icehockey
12.6		Yes	Artistic gymnastics	4.8	Artistic gymnastics	14.9		Yes	Fall at same level	4.3	Air acrobatics
12.7	Yes		Artistic gymnastics	2.2	Artistic gymnastics	15.2 [c]	Yes	Yes	Skateboarding	5.7	Icehockey
13.4	Yes	Yes	Artistic gymnastics	1.0	Artistic gymnastics	15.2			American football	2.0	American football
13.5		Yes	Cheerleading	3.2	Cheerleading	15.3	Yes	Yes	Running	5.0	Dance
13.6	Yes	Yes	Fall from height	1.4	Gym	15.4 [c]	Yes		Trampoline	2.7	Trampoline gymnastics
14.1		Yes	Cheerleading	1.4	Cheerleading	15.5	Yes		Piching	1.6	Gym
14.8	Yes		Football	1.3	Football	15.6		Yes	Taekwando	3.5	Gym
15.8			Fall from height	4.3	Gym	15.9			Trampoline	5.2	Piano
15.8			Fall from height	4.3	Gym						

[a] Pin fixation
[b] Anchor fixation
[c] Patients who quit their sport due to the injury.
[d] Patients who continued sports less strenuous for the injured elbow.
